# Pharmacists’ communication skills with deaf and hard of hearing patients: A needs assessment

**DOI:** 10.1371/journal.pone.0286537

**Published:** 2023-06-29

**Authors:** Noha Al Aloola, Maram Alanazi, Norah Alotaibi, Monira Alwhaibi

**Affiliations:** 1 Clinical Pharmacy Department, College of Pharmacy, King Saud University, Riyadh, Saudi Arabia; 2 Medication Safety Research Chair, College of Pharmacy, King Saud University, Riyadh, Saudi Arabia; 3 College of Pharmacy, King Saud University, Riyadh, Saudi Arabia; Lamar University, UNITED STATES

## Abstract

**Objectives:**

Assess the Saudi pharmacists’ perceptions of their responsibilities toward deaf and hard-of-hearing (DHH) patients, their current practices, and their need for communication skills training.

**Study design:**

A prospective cross-sectional study.

**Methods:**

Pharmacist and deaf communication questionnaire (PDCQ), a newly structured, validated, pilot-tested, and self-administered online questionnaire, was used to collect data. A total of 303 pharmacists working in the Saudi community and outpatient pharmacies participated in the study. Data were analyzed using SPSS and descriptive statistics were used to describe the study findings. These included Mean ± standard deviation (SD), frequency, and Chi-square tests.

**Results:**

Most pharmacists perceived that DHH patients had difficulty correctly understanding their medication instructions. Writing was the most common method used for communication, whereas the unavailability of interpreters and the low reading levels of these patients were the highest barriers to communication. Moreover, most pharmacists believed that they should be skilled at communicating with DHH patients. However, many pharmacists felt that they are not well prepared to communicate with these patients.

**Conclusion:**

This research highlights the poor skills, low confidence and low knowledge Saudi pharmacists have about their legal obligations towards DHH patients. In addition, there is paucity of sufficient resources to help pharmacists improve their communication with such patients.

## Introduction

The World Health Organization (WHO) defines people with profound hearing loss as deaf, whereas those with mild to severe hearing loss are classified as Hard of Hearing (HOH) people [1]. Approximately 466 million people worldwide have disabling hearing loss; this number is estimated to exceed 900 million by 2050 (i.e., one in every ten people) [1]. In Saudi Arabia, a disability survey by the General Authority of Statistics showed that approximately 1.4% of the Saudi population has disabling hearing loss, with 0.4% having deafness [2].

Studies have shown that deaf people have low health literacy, owing to communication barriers with healthcare providers [3]. In addition, studies have indicated that this communication barrier negatively affects deaf people’s physical and mental health, as they can face prolonged or unnecessary illnesses due to inadequate communication with their healthcare providers [4, 5].

Several methods by which healthcare providers, including pharmacists, can communicate with DHH patients include using Sign Language, speech-reading or lip-reading, writing notes, using interpreters and visual aids, mobility aids, and telecommunication devices for the deaf (TCD) [4–9]. These are special telecommunications equipment used by people who cannot use a regular telephone because of hearing loss or speech impairment, such as teletypewriters (TTY) and videophones for the deaf (VP). Studies have indicated a lack of awareness among healthcare providers, including pharmacists, regarding their legal obligations toward DHH patients [10, 11]. A survey evaluating pharmacists’ practices involving deaf patients in the United States showed that most pharmacists perceived their legal obligations to deaf patients to be limited to providing written information [10]. Another study in Brazil showed that the majority of family health professionals, including pharmacists, were aware of Sign Language; however, none used it to communicate with deaf patients [12].

Pharmacists play an important role in improving the medication literacy of DHH patients [13]. Based on the ASHP guidelines for pharmacists conducting patient education and counseling, pharmacists should assess the patient’s sensory status in addition to their cognitive abilities, learning style, and physical status before educating the patient [14]. However, pharmacists perceive deafness and hearing difficulties as barriers to patient counseling [15, 16]. Moreover, DHH patients indicated a lack of understanding of their medication instructions and dependence on their children, spouses, or friends for help with such information. In addition, some prefer ordering refills online, using a mail-service pharmacy, or using a drive-through window to avoid frustration and direct contact with pharmacists [17].

Teaching pharmacy students about their legal obligations toward DHH patients and preparing them to communicate effectively with them is crucial. Some universities have incorporated these competencies into their curriculum [18, 19]. The World Federation of the Deaf (WFD) conducts several training programs and projects to ensure that deaf people across the globe are equipped with knowledge, tools, and strategies to advocate for their rights. These include recognition of and respect for their cultures and Sign Languages. Moreover, the WFD has initiated a health resource initiative. The project consists of different parts, the first of which contains a survey on health issues for deaf leaders and organization members. The second part contains a survey on deaf communities and encourages members to become involved with their health employees through a website [20].

In Saudi Arabia, several initiatives have been implemented to support DHH patients. In 2019, the Ministry of Public Health launched the “We Are with You” initiative [21]. This initiative aims to improve (i) the communication between deaf patients and healthcare providers, (ii) the knowledge of deaf people about their health rights, and (iii) the use of Sign Language in healthcare services to enhance the healthcare services at the Ministry of Health (MOH) facilities [21]. Moreover, the MOH approved the use of Sign Language in health services provided to pilgrims, in addition to providing several Sign Language training courses for health practitioners to improve their communication with deaf people [22]. However, there is a lack of research assessing Saudi pharmacists’ perceptions of their responsibilities toward DHH patients, their current practices, and their need for communication skills training. This study aimed to assess the need for improving pharmacists’ communication skills with DHH patients, from the perspective of pharmacists.

## Methods

### Study design and sample

This prospective cross-sectional study used self-administered online questionnaire targeting pharmacists working in community and outpatient pharmacies in the Kingdom of Saudi Arabia (KSA).

### Questionnaire

Currently, there is no validated instrument for exploring pharmacists’ perceptions of the need for communication skills with patients who are DHH. A questionnaire was developed based on literature review. It consisted of two main parts: (1) general information and (2) perception of the need for education on communicating with DHH people. This included assessing pharmacists’ perceptions of their roles in the communication and education of DHH patients, exploring their experiences and current practices (including methods used for communication and the resources available to them), assessing their perspectives toward adopting these skills, and their preferences regarding such education.

To ensure face and content validity, a group of investigators reviewed the survey for relevance, content, clarity, and ease of understanding. Each item was linked to the objectives of the study. The survey was pilot tested on a purposive sample of pharmacists (n = 20) to evaluate the appropriateness of the questions for the target sample. The comments and suggestions of participants were considered. The frequency distribution (frequency of endorsement) was analyzed to determine the proportion of unanswered items that were not understandable or interpretable. There were no questions with low response rate. The internal consistency of the items was estimated using Cronbach’s alpha. A reliability analysis of the perception items yielded a Cronbach’s alpha coefficient of 0.7.

### Procedure

The questionnaire link and a participant information statement explaining the purpose of the study were posted on social media such as WhatsApp and Twitter using pharmacists’ groups between December 2020 and January 2021, asking any pharmacist in KSA working at community pharmacies or outpatient pharmacies to participate.

### Statistical analysis

Data were analyzed using Statistical Package for the Social Sciences (SPSS) software. Descriptive statistics were used to describe the findings. Mean ± standard deviation (SD) and frequency (%) were used to describe continuous and categorical variables. Chi-square tests were used to conduct bivariate analyses of categorical and continuous variables. Statistical significance was set at p < 0.05.

### Ethical approval

This study conformed to the ethical guidelines of and was approved by the King Saud University Institutional Review Board (KSU-IRB 017E). Participants were asked to complete an informed consent form before completing the online survey. The information statement explained the purpose of the study, highlighted voluntary involvement, and discussed the right to withdraw from the study at any time before completing the anonymous survey.

## Results

A total of 303 pharmacists participated in the study; 45% (n = 137) were male, and 55% (n = 166) were female (Table 1). Approximately 40% of the participating pharmacists worked in community pharmacies, whereas 60% worked in outpatient or emergency pharmacies. All had varying years of experience and exposure to DHH patients (approximately 50% of pharmacists had seen DHH patients in the previous month). Some pharmacists reported hearing difficulties (9.6%, n = 29), while others used hearing aids (5.3%, n = 16). In addition, 9.2% (n = 28) of the pharmacists had a deaf family member, and 10.6% (n = 32) had a family member who was HOH. Written notes (25%, n = 15), Sign Language (38%, n = 23), and lip reading (37%, n = 22) were employed to communicate with such DHH family members.

**Table 1:** Characteristics of the study sample.

(n = 303)		N (%)
**Gender**	Male	137 (45.2%)
	Female	166 (54.8%)
**Nationality**	Saudi	271 (89.4%)
	Non-Saudi	32 (10.6%)
**Work**	Community pharmacy	121 (39.9%)
	Hospital (outpatient/ER)	182 (60.1%)
**Years of work experience**	Less than 1 year	115 (38.0%)
	1–5 years	126 (41.6%)
	6–10 years	31 (10.2%)
	More than 10 years	31 (10.2%)
**Have hearing difficulty**	Yes	29 (9.6%)
	No	274 (90.4%)
**Using hearing aids**	Yes	17 (5.6%)
	No	12 (3.96%)
**Have a family member who has hearing difficulty**	Yes, Deaf	28 (9.2%)
	Yes, HOH	32 (10.6%)
	No	243 (80.2%)
**Communication with family member who has hearing difficulty (n = 60)**	Written notes	15 (25%)
	Sing language	23 (38%)
	Lips reading	22 (37%)
**Number of deaf or HOH patients seen last month**	None	150 (49.5%)
	1–3	133 (43.9%)
	More than 3	20 (6.6%)

### Current practice and communication barriers

**Fig 1:**
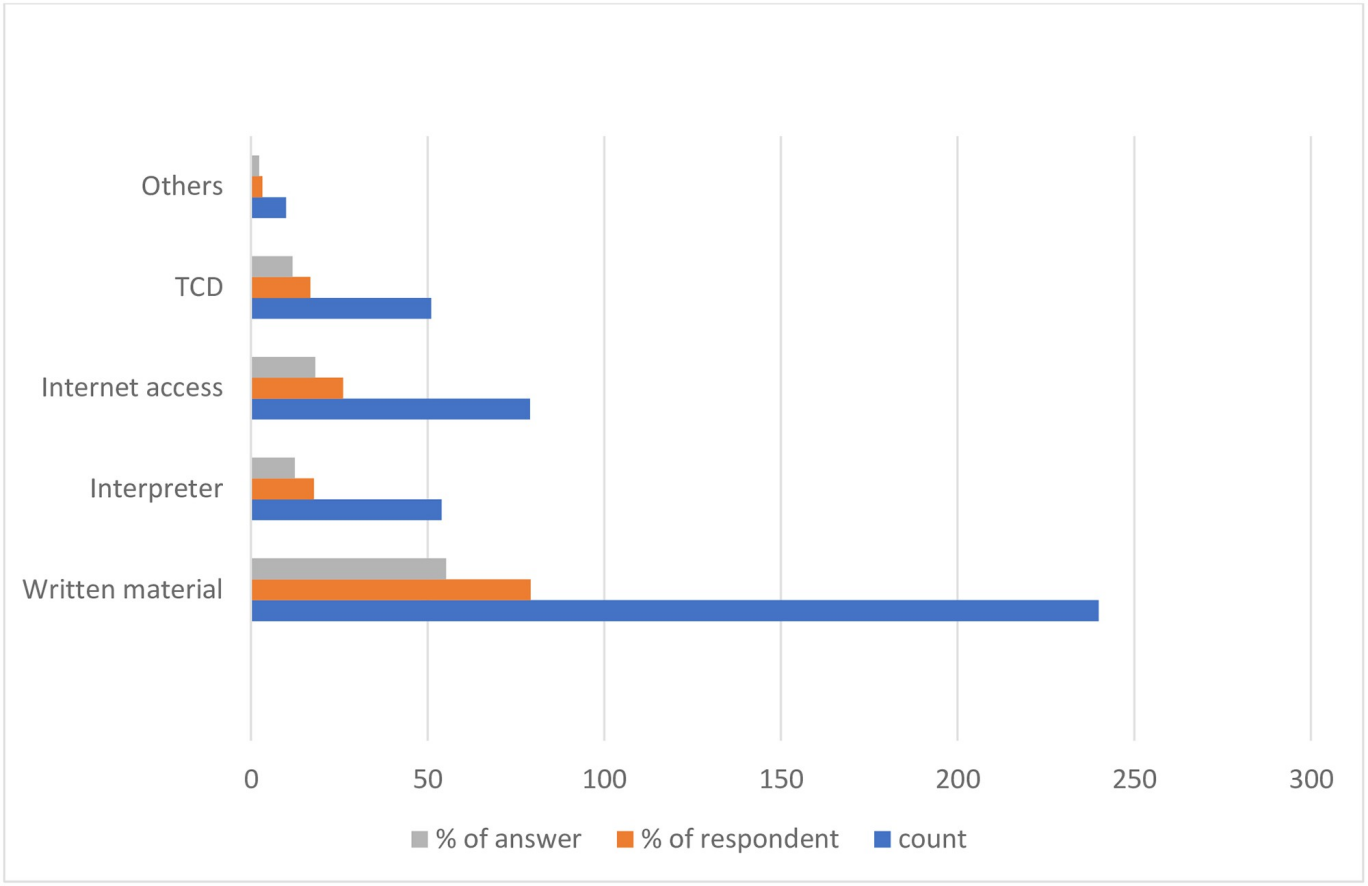
Resources accessible to pharmacist for communication with DHH patients. *depend on using louder voice or pronounce words clearly, basics knowledge in Sign Language, using pictures and signs.

**Fig 2:**
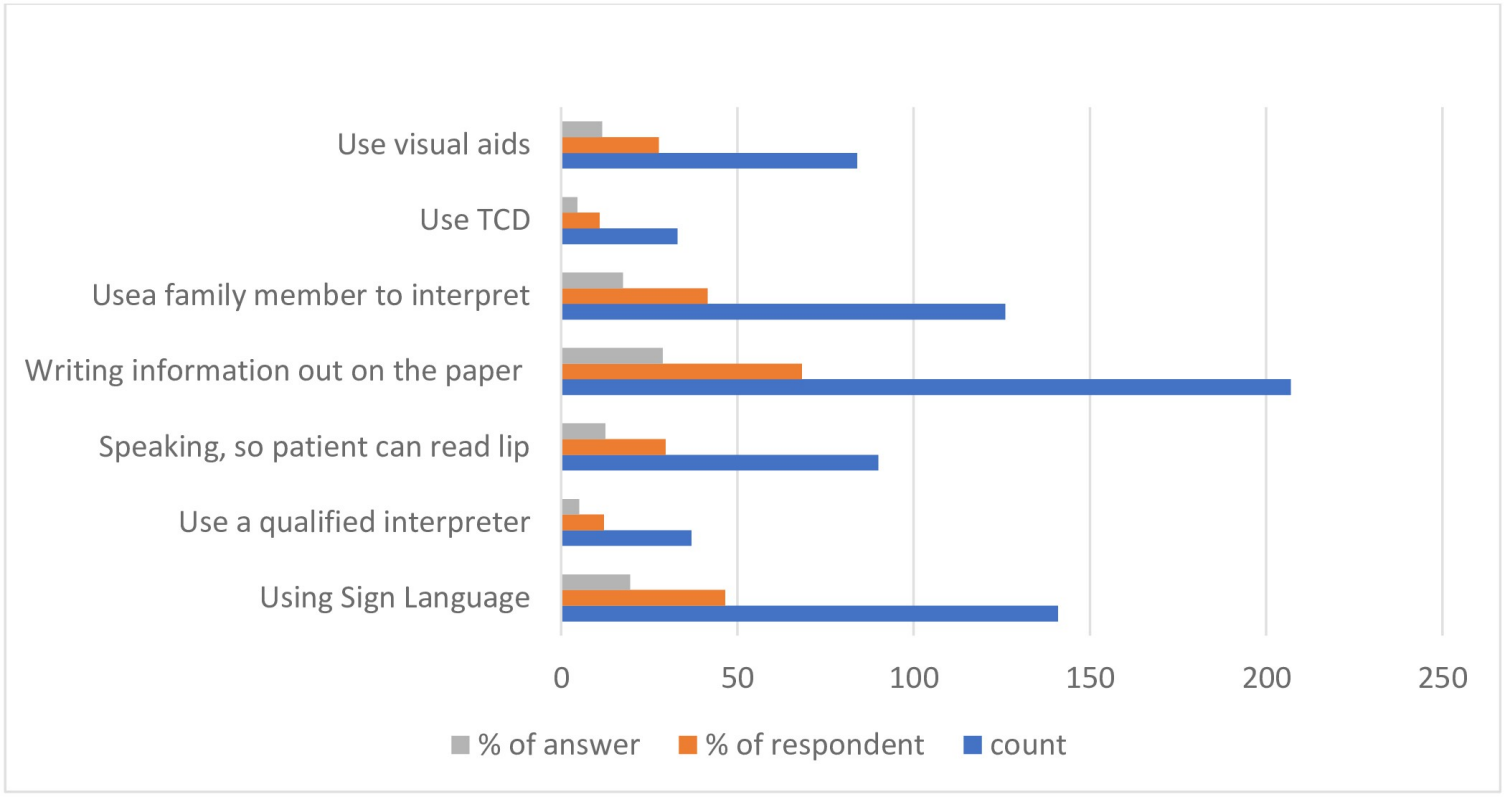
Methods used by pharmacists to communicate with DHH patients.

**Fig 3:**
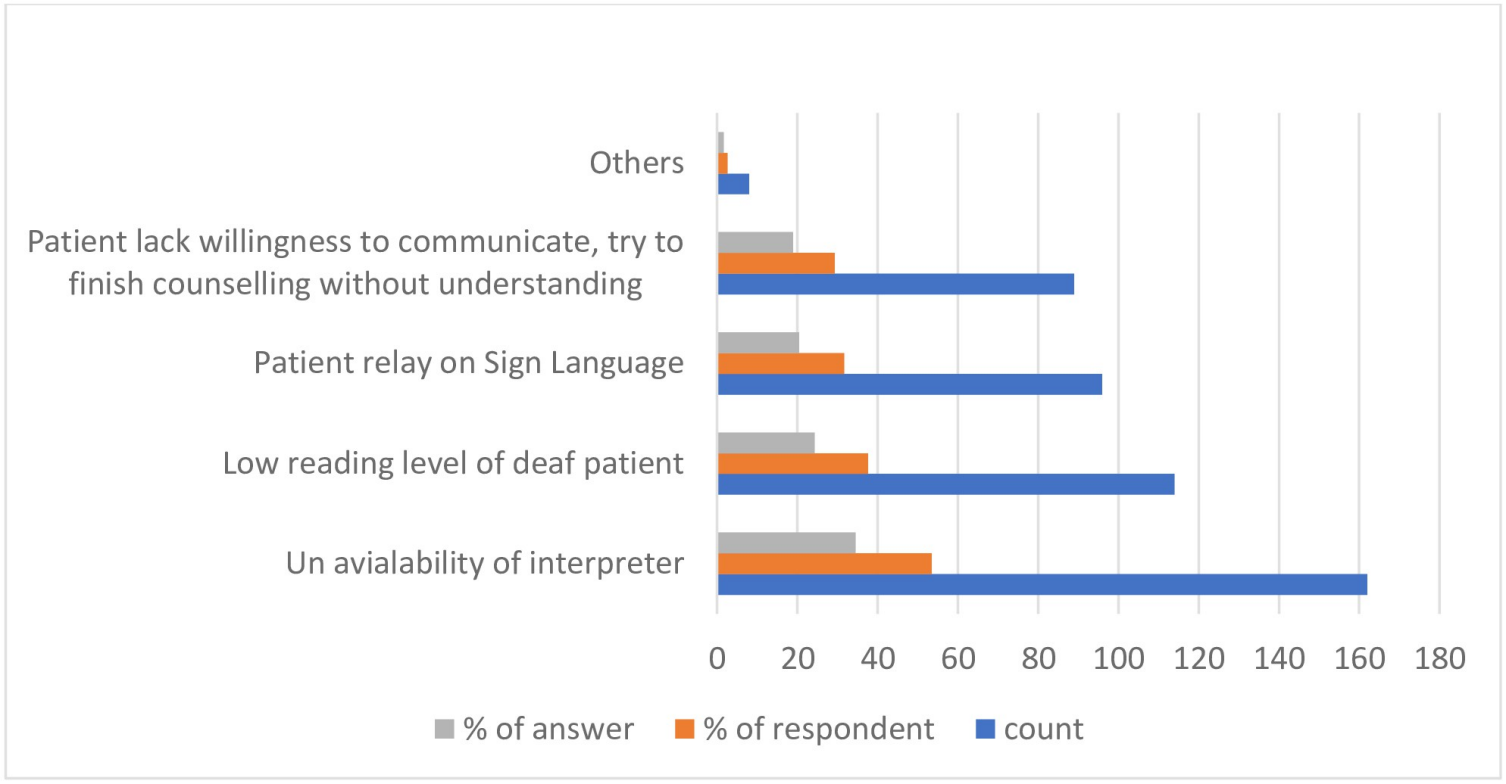
Barriers of communication with DHH patients. * lack of pharmacists’ knowledge about sign language, patients having multiple difficulties and did not have a caregiver, difficulty in reading lips of female pharmacists because of their face cover.

The most accessible resources for pharmacists to communicate with DHH patients were written materials (55.3%) and the Internet (18.2%). This was followed by interpreters (12.4%) and TCD (11.8%). Few participants (2.3%) relied on using a loud voice, pronouncing words clearly, using basic knowledge of Sign Language, or using pictures and signs (Fig 1). The pharmacists mainly used written materials (28.8%), Sign Language (19.6%), and a family member to interpret (17.5%), while communicating with DHH patients. These were followed by speaking clearly so that the patient could read the lips (12.5%), using visual aids (11.7%), using a qualified interpreter (5.2%), and using a TCD for the Deaf (4.6%) (Fig 2). The common barriers pharmacists faced when communicating with DHH patients were the unavailability of an interpreter (34.5%), low reading levels of deaf patients (24.3%), patients’ reliance on Sign Language (20.5%), and patients’ lack of willingness to communicate (18.98%). Other barriers reported (1.7%) included lack of pharmacists’ knowledge of Sign Language, patients having multiple difficulties but no caregivers, and difficulty reading the lips of female pharmacists because of their face coverings (Fig 3).

### Previous education of participating pharmacists

This study found that only 26% (n = 80) of participating pharmacists had previously received education on communication with DHH patients. Moreover, there were variations in the content of this education, the sites where it was received, the duration of education and the specialization of the people who provided that education. The majority were only involved in training on the use of Sign Language (61.25%, n = 49), whereas others provided instructions on how to effectively use body gestures (33.75%, n = 27), write notes (18.75%, n = 15), use visual aids (12.5%, n = 10), and use TCD for the deaf (5%, n = 4). The most commonly reported educational sites were universities, which conducted curricular courses (28.75%; n = 23) and extracurricular activities (11.25%; n = 9). This was followed by hospitals (30%, n = 24), social meetings (8.75%, n = 7), online education (18.75%, n = 15), and community pharmacies (2.5%, n = 2). Most of this education was provided over one to two days (36.25%; n = 29) and was provided by pharmacists (40%; n = 32). Additionally, a low percentage of pharmacists (34%; n = 103) knew about “We Are with You” (an initiative by MOH that provides a Sign Language training program for healthcare providers), and around 65% (n = 67) of them had participated in this training program (Table 2).

**Table 2:** Previous education of participating pharmacists on skills required for communicating with DHH patients.

(n = 303)		N (%)
**Received previous education**	Yes	80 (26.4%)
	No	223 (73.6%)
**Content**	Only sign language	49 (61.25%)
	Writing notes	15 (18.75%)
	Visual aids	10 (12.5%)
	TDC	4 (5%)
	Body gestures	27 (33.75%)
**Site**	University curricular	23 (28.75%)
	Extra-curricular activity	9 (11.25%)
	Hospital	24 (30%)
	Social meeting	7 (8.75%)
	Online	15 (18.75%)
	Other working place	2 (2.5.%)
**Duration**	Half day	29 (36.25%)
	1–2 days	29 (36.25%)
	One week	17 (21.25%)
	Others (3 weeks, 2 months)	4 (5%)
**Specialty of education provider**	Pharmacists	32 (40%)
	Physicians	11 (13.75%)
	Health education	9 (6.6%)
	Special need specialist	18 (22.5%)
	Not know	10 (12.5%)

### Need for education

This study showed that approximately 50% (n = 152) of the enrolled pharmacists were confident and felt adequately prepared to communicate with and educate DHH patients. This was owing to their previous education on communication skills required for dealing with DHH patients (p-value <0.001). Additionally, most pharmacists (69%; n = 209) perceived that DHH patients had difficulty understanding their medication instructions correctly, and this perception was significantly related to the pharmacists’ place of work, where hospital pharmacists had higher perceptions than community pharmacists (p = 0.032). In addition, 33.7% (n = 102) of the pharmacists believed that DHH patients did not use their medications correctly, and this perception was significantly related to having a family member with hearing difficulty (p = 0.016) (Table 3).

**Table 3:** Pharmacists’ confidence, and perception of need for development of communication skills with DHH people.

		Do you feel you are adequately prepared to communicate and educate patients with DHH about their medications?			Do you think that DHH people facing difficulties in understanding their medications instructions provided by pharmacists?			Do you think that DHH people use their medications provided by pharmacists correctly?		
		Yes N (%)	No N (%)	P-value	Yes N (%)	No N (%)	P-value	Yes N (%)	No N (%)	P-value
**Place of work**	Community pharmacy	68 (56.2%)	53 (43.8%)	0.087	75 (62%)	46 (38%)	**0.032**	77 (63.6%)	44 (36.4%)	0.417
	Hospital (outpatient/ER)	84 (46.3%)	98 (53.8%)		134 (73.6%)	48 (26.4%)		124 (68.1%)	58 (31.9%)	
**Years of work experience**	Less than 1 year	58 (50.4%)	57 (49.6%)	0.547	80 (69.6%)	35 (30.4%)	0.807	76 (66.1%)	39 (33.9%)	0.431
	1–5 years	59 (46.8%)	67 (53.2%)		88 (69.8%)	38 (30.2%)		79 (62.7%)	47 (37.3%)	
	6–10 years	16 (51.6%)	15 (48.4%)		22 (71%)	9 (29%)		22 (71%)	9 (29%)	
	More than 10 years	19 (61.3%)	12 (38.7%)		19 (61.3%)	12 (38.7%)		24 (77.4%)	7 (22.6%)	
**Have hearing difficulty**	Yes	24 (82.8%)	5 (17.2%)	**<0.001**	23 (79.3%)	7 (20.7%)	0.206	22 (75.9%)	(7) 24.1%	0.254
	No	128 (46.7%)	146 (53.3%)		186 (67.9%)	88 (32.1%)		179 (65.3%)	95 (34.7%)	
**Have any family member who has hearing difficulty**	Yes, Deaf	25 (89.3%)	3 (10.7%)	**<0.001**	24 (85.7%)	4 (14.3%)	0.111	25 (89.3%)	3 (10.7%)	**0.016**
	Yes, hard of hearing	20 (62.5%)	12 (37.5%)		23 (71.9%)	9 (28.1%)		23 (71.9%)	9 (28.1%)	
	No	107 (44%)	136 (56%)		162 (66.7%)	81 (33.3%)		153 (63%)	90 (37%)	
**Number of DHH patients seen last month**	None	71 (47.3%)	79 (52.7%)	**0.066**	103 (68.7%)	47 (31.3%)	0.625	102 (68%)	48 (32%)	0.100
	1–3	66 (49.6%)	67 (50.4%)		94 (70.7%)	39 (29.3%)		82 (61.7%)	51 (38.3%)	
	More than 3	15 (75%)	5 (25%)		12 (60%)	8 (40%)		17 (85%)	3 (15%)	
**Previous education about communication skills with people having DHH**	Yes	62 (77.5%)	18 (22.5%)	**<0.001**	50 (62.5%)	30 (37.5%)	0.144	55 (68.8%)	25 (31.3%)	0.594
	No	90 (40.4%)	133 (49.8%)		159 (?71.3%)	64 (28.7%)		146 (65.5%)	77 (34.5%)	

However, 61.7% (n = 187) of the pharmacists perceived that they should be skilled and able to communicate with these patients, while others thought that they had no legal obligation toward these patients (12.5%; n = 38) or that their legal obligation was limited to the provision of written materials (10.6%; n = 32). This perception was related to the pharmacists’ experience (p = 0.041) of having hearing difficulty (p <0.001), having a family member with hearing difficulty (p <0.001), and previous education on communication skills’ development with those patients (p <0.001) (Table 4). However, 63.7% (n = 193) of the pharmacists indicated their need for a training program on using Sign Language to improve their communication with DHH patients. Some pharmacists also suggested using interactive materials (e.g., adding medication information to a video, uploading it to the Internet, and assigning a barcode that the patient can use).

**Table 4:** Pharmacists’ perception of their legal obligation towards DHH patients.

		Do you think that communicating with patients having DHH about their medications is the responsibility of a pharmacist?				
		No legal obligation is present for pharmacist N (%)	Pharmacist should be able and skilled to communicate with these patients N (%)	An interpreter should be available to help in communication with these patients N (%)	Providing written materials will be enough N (%)	P-value
**Place of work**	Community pharmacy	14 (11.6%)	75 (62%)	11.6% (14)	18 (14.9%)	0.143
	Hospital pharmacy (outpatient/ER)	24 (13.2%)	112 (61.5%)	17.6% (32)	14 (7.7%)	
**Experience**	Less than 1 year	10.4% (12)	68.7% (79)	13% (15)	7.8% (9)	**0.041**
	1–5 years	16.7% (21)	57.1% (72)	16.7% (21)	9.5% (12)	
	6–10 years	9.7% (3)	64.5% (20)	19.4% (6)	6.5% (2)	
	More than 10 years	6.5% (2)	51.6% (16)	12.9% (4)	29% (9)	
**Have hearing difficulty**	Yes	37.9% (11)	44.8% (13)	13.8% (4)	3.4% (1)	**<0.001**
	No	9.9% (27)	63.5% (174)	15.3% (42)	11.3% (31)	
**Have any family member who has hearing difficulty**	Yes, Deaf	39.3% (11)	53.6% (15)	7.1% (2)	0% (0)	**<0.001**
	Yes, hard of hearing	21.9% (7)	65.6% (21)	12.5% (4)	0% (0)	
	No	8.2% (20)	62.1% (151)	16.5% (40)	13.2% (32)	
**Number of DHH patients seen last month**	None	12.7% (19)	62% (93)	12% (18)	13.3% (20)	0.252
	1–3	10.5% (14)	63.2% (84)	18.8% (25)	7.5% (10)	
	More than 3	25% (5)	50% (10)	15% (3)	10% (2)	
**Previous education about communication skills with people having DHH**	Yes	27.5% (22)	60% (48)	7.5% (6)	5% (4)	**<0.001**
	No	7.2% (16)	62.3% (139)	17.9% (40)	12.6% (28)	

Furthermore, the majority of pharmacists (81%; n = 246) showed willingness to participate in a communication skills program so as to better communicate with DHH patients if it was offered by their institution, and around 66% (n = 200) of them believed that attending such education should be mandatory for all pharmacists and should be provided over several days to one week. Moreover, most pharmacists (81%, n = 245) agreed that pharmacy students needed to be educated and prepared to communicate with these patients.

## Discussion

This was the first study to evaluate the need for Saudi pharmacists to be educated on skills required for communication with DHH patients and to explore their perceptions, practices, and experiences. The results showed that pharmacists in Saudi Arabia have less awareness of their legal obligations toward DHH patients and have poor competencies to communicate with these patients. However, the pharmacists indicated their need for education on developing skills required for communication with DHH patients and their willingness to participate in such education.

This study revealed that DHH patients in the country lack the willingness to communicate and try to complete counseling with pharmacists without understanding. This behavior was also reported in a previous study in which DHH patients depended on their friends and children for help with medications and tended to avoid direct interactions with pharmacists [17]. This is an important finding, since literature indicates the poor health literacy of deaf patients to be associated with inadequate communication with their healthcare providers, which negatively affects their overall health [3–5].

Another issue this study found was that, despite the fact that around half of the pharmacists had seen DHH patients in the previous month, with the low reading level of some deaf patients, and patients’ reliance on Sign Language, the majority of pharmacists relied on using written notes to communicate with DHH patients, and only few used Sign Language. The same issue was reported in Brazil, where poor use of Sign Language by pharmacists was reported despite their awareness of it [12]. However, in our study, we cannot co-relate the poor use of Sign Language by the pharmacists to the lack of knowledge that pharmacists have, since some pharmacists do have the knowledge but patients lack the willingness to communicate or do not know to communicate through Sign Language. These factors should be investigated further in future studies.

Moreover, although most of the participating pharmacists believed that DHH patients faced difficulties in understanding their medication instructions provided by pharmacists, some pharmacists perceived that there was no legal obligation on their part toward these patients or that their legal obligation was limited to the provision of written materials. This is consistent with previous studies conducted in the United States, where most pharmacists perceived that their legal obligations to deaf patients were limited to the provision of written information [10, 11]. Additionally, despite the fact that most pharmacists perceived that DHH patients had difficulty understanding their medication instructions correctly, and that most of them believed that they should be skilled and able to communicate with these patients; this study found that around half of the enrolled pharmacists lacked confidence and felt they were not adequately prepared to communicate with and educate these patients. However, they were willing to participate in skills programs for improving their communication with DHH patients. Furthermore, only a few pharmacists had received previous education on communicating with these patients, which necessitates the need for such education.

This study provides a picture of current pharmacists’ practices regarding communication with hearing-impaired patients in the country, assesses their perceptions of their legal obligations toward these patients, and evaluates their need for education on communication skills with hearing-impaired patients. The study has some limitations: As the survey was self-reported, recall bias cannot be excluded. In addition, the response rate for our survey is unknown, as we used social media to advertise the survey (with a survey link). Therefore, we do not know how many potential participants came across the survey information and decided not to respond. In addition, the exact percentage of pharmacists participating from each region in the country is unknown. However, we believe that our results are representative because the questionnaire was distributed on different social media platforms.

## Conclusion

This study describes the need of pharmacists to educate themselves for better communication with DHH patients. The results of this study can be used to help develop an educational program for pharmacists regarding skills on communication with DHH patients. This program should target at improving pharmacists’ knowledge of their legal obligations toward these patients apart from developing their communication skills.

## Supporting information

S1 Data (XLSX)

S1 File (DOCX)

S1 Raw images (PDF)
