# Supplementary material for: Pharmacists’ communication skills with deaf and hard of hearing patients: A needs assessment
Source: PLoS One. 2023 Jun 29;18(6):e0286537. doi: 10.1371/journal.pone.0286537 (PMC10310020; doi:10.1371/journal.pone.0286537)
Supplement: S1 File — (DOCX) [file pone.0286537.s002.docx]

**Pharmacist and deaf communication questionnaire (PDCQ)**

**GENERAL INFORMATION**

**Part 1. This section requests you to provide general information about yourself. This is important because different pharmacist may have different experiences and requirements for communication with deaf and hard of hearing patients, and we wish to understand individual needs.**

**In the table below, please place a tick in the box next to the most suitable response.**

| 1. What is your gender? | ☐ Male.  ☐ Female. |
| --- | --- |
| 1. What is your nationality | ☐ Saudi.  ☐ Non-Saudi. |
| 1. Where is your current work? | ☐ Community pharmacy.  ☐ Hospital (outpatient/ER) |
| 1. For how many years have you been work as pharmacist (experience)? | ☐ Less than 1 year  ☐ 1-5 years.  ☐ 6-10 years.  ☐ More than 10 years. |
| 1. Do you have hearing difficulty? | ☐ Yes.  ☐ No. |
| 1. **If you answer 5 yes**, do you use hearing aids? | ☐ Yes.  ☐ No. |
| 1. Do you have any family member who has hearing difficulty or been diagnosed as deaf person ? | ☐ Yes, Deaf.  ☐ Yes, hard of hearing.  ☐ No. |
| 1. **If you answer 7 yes**, how do you communicate with him/her? | ☐ Written notes.  ☐ Sing language.  ☐ Lips reading. |
| 1. How many deaf or hard of hearing patients have you seen last month? | ☐ None  ☐ 1-3  ☐ More than 3 |
| 1. Generally, what methods do you use to communicate with deaf and hard of hearing patients?   (**You can choose more than one**). | ☐ Use Sign Language.  ☐ Use a qualified interpreter.  ☐ Speaking so the patient can read your lips.  ☐ Writing information out on the paper for the patient.  ☐ Use a family members to interpret.  ☐ Use of telecommunication devices for the deaf.  ☐ Use visual aids  ☐ Other, Please write……………. |
| 1. What are the resources accessible for you to communication with deaf patients?   (**You can choose more than one**). | ☐ Written material.  ☐ Interpreters.  ☐ Internet access.  ☐ Telecommunication devices.  ☐ Other, Please write……………. |
| 1. What barriers did you faced when you communicate with deaf and hard of hearing patients?   (**You can choose more than one**) | ☐ Unavailability of an interpreter.  ☐ low reading level of deaf patient.  ☐ Patient relies on Sign Language.  ☐ Patient lack of willingness to communicate, trying to finish counselling without understanding.  ☐ Other, Please write……………. |

**PERCEPTION OF NEED FOR EDUCATION**

**Part 2. Education about communication with deaf and hard of hearing people implies some training/program/curriculum part that covered the basic skills and methods. We would like to ask you about your experience, your thoughts about being provided with such education, and opportunity that should be available to you.**

**In the table below, please place a tick in the box next to the most suitable response.**

| 1. Do you think that deaf and hard of hearing people use their medications provided by pharmacists correctly ? | ☐ Yes.  ☐ No. |
| --- | --- |
| 1. Do you think that deaf and hard of hearing people facing difficulties in understanding their medications instructions provided by pharmacists? | ☐ Yes.  ☐ No. |
| 1. Do you think that communicating with deaf and hard of hearing people about their medications is a responsibility of pharmacist? | ☐ No legal obligation is present for pharmacist.  ☐ Pharmacist should be able and skilled to communicate with these patients.  ☐ An interpreter should be available to help in communication with these patients  ☐ Providing written materials will be enough. |
| 1. What do you need to improve your communication with deaf and hard of hearing patients? | ☐ Nothing as it is not my responsibility  ☐ Nothing providing written materials will be enough.  ☐ A training program for using Sign Language.  ☐ Other, Please write……………. |
| 1. Do you received any form of education about communication skills with deaf and hard of hearing people previously? | ☐ Yes.  ☐ No. **If No, please go directly to the question 9.** |
| 1. **If yes**, where did you receive it? | ☐ University, curricular course  ☐Extra-curricular activity in university  ☐ Hospital.  ☐ Social meeting.  ☐ Online.  ☐ Other ................. |
| 1. Who conducted that education? | ☐ Physicians.  ☐ Pharmacists.  ☐ Health educators.  ☐ Nurses.  ☐ Special need specialists.  ☐ I don’t know. |
| 1. What was the content of that course or program? (**You can choose more than one**) | ☐ Only how to use Sign language  ☐ How to effectively engage those patients in communication using body gesture.  ☐ How to use writing notes  ☐ How to use visual aids  ☐ How to use telecommunication device for deaf (TDD)  ☐ Other ................. |
| 1. What was the duration of education training? | ☐ Half day  ☐ 1- 2 days  ☐ One weeks.  ☐ Other , ………… |
| 1. Do you feel you are adequately prepared to communicate and educate patients with deafness or hard of hearing about their medications? | ☐ Yes  ☐ No |
| 1. If pharmacists in your institute be offered an opportunity to undertake education in communication and counseling skills with deaf and hard of hearing people, would you be willing to participate? | ☐ Yes  ☐ No |
| 1. Do you think attending education should be mandatory for all pharmacists or not? | ☐ Yes  ☐ No |
| 1. For how long do you think this training should be? | ☐ One day activity  ☐ Several days- a week activity  ☐ More than week |
| 1. Do you believe that pharmacy students’ need to be educated and prepared for communication with deaf and hard of hearing people? | ☐ Yes.  ☐ No. |
| 1. Did you hear about “**We Are With You**” an initiative by Ministry of public health, that provide Sign Language training programs for healthcare providers? | ☐ Yes.  ☐ No. |
| 1. If yes, did you participate in this program? | ☐ Yes.  ☐ No. |

If you have any suggestions for the content of such education, please write them out here:

...............................................................................................................................................................................................................................................................................................................................................................................................................................................................................................................................................................................................................................................

Thank you
