# Supplementary material for: Pharmacists’ communication skills with deaf and hard of hearing patients: A needs assessment
Source: PLoS One. 2023 Jun 29;18(6):e0286537. doi: 10.1371/journal.pone.0286537 (PMC10310020; doi:10.1371/journal.pone.0286537)
Supplement: S1 Raw images — (PDF) [file pone.0286537.s003.pdf]

Figure1: Resources accessible to pharmacist for communication with DHH patients

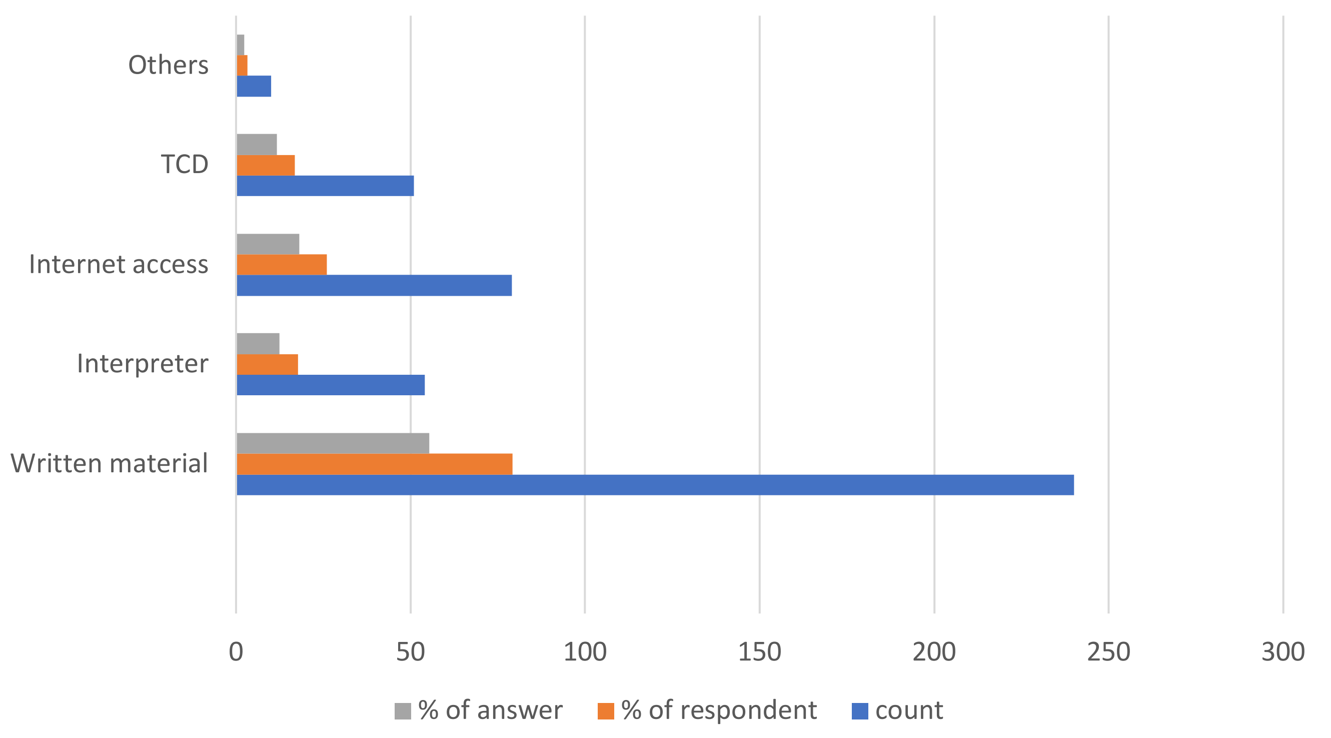

* depend on using louder voice or pronounce words clearly, basics knowledge in Sign Language, using pictures and signs.

Figure2: Methods used by pharmacists to communicate with DHH patients

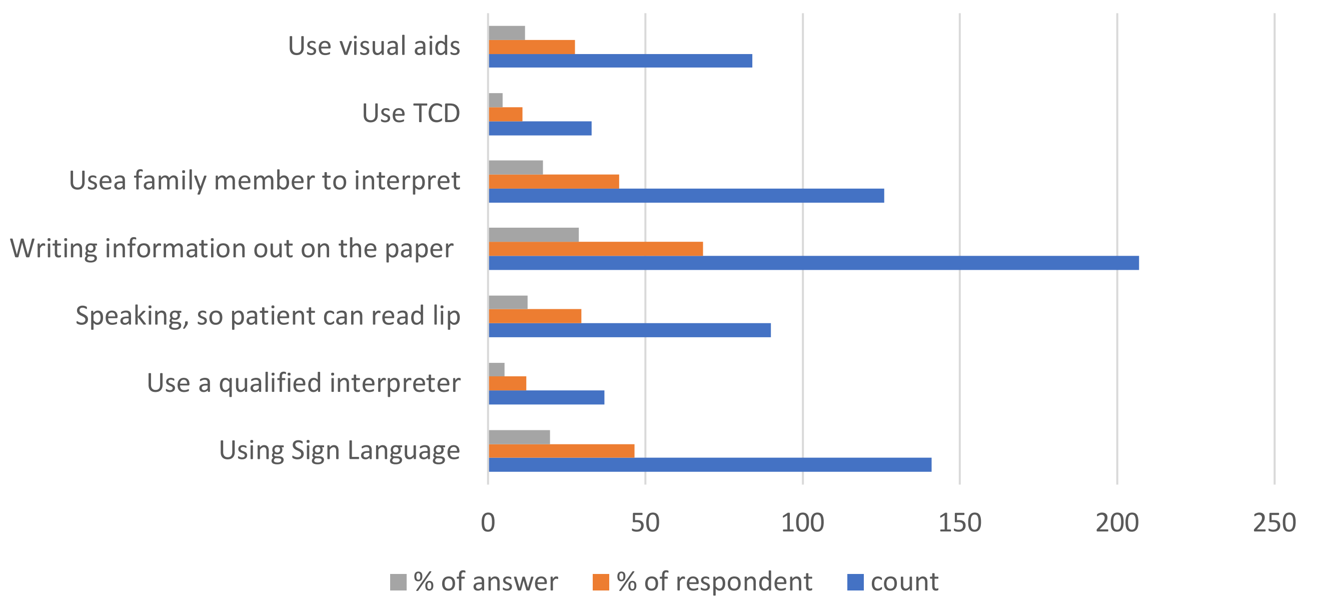

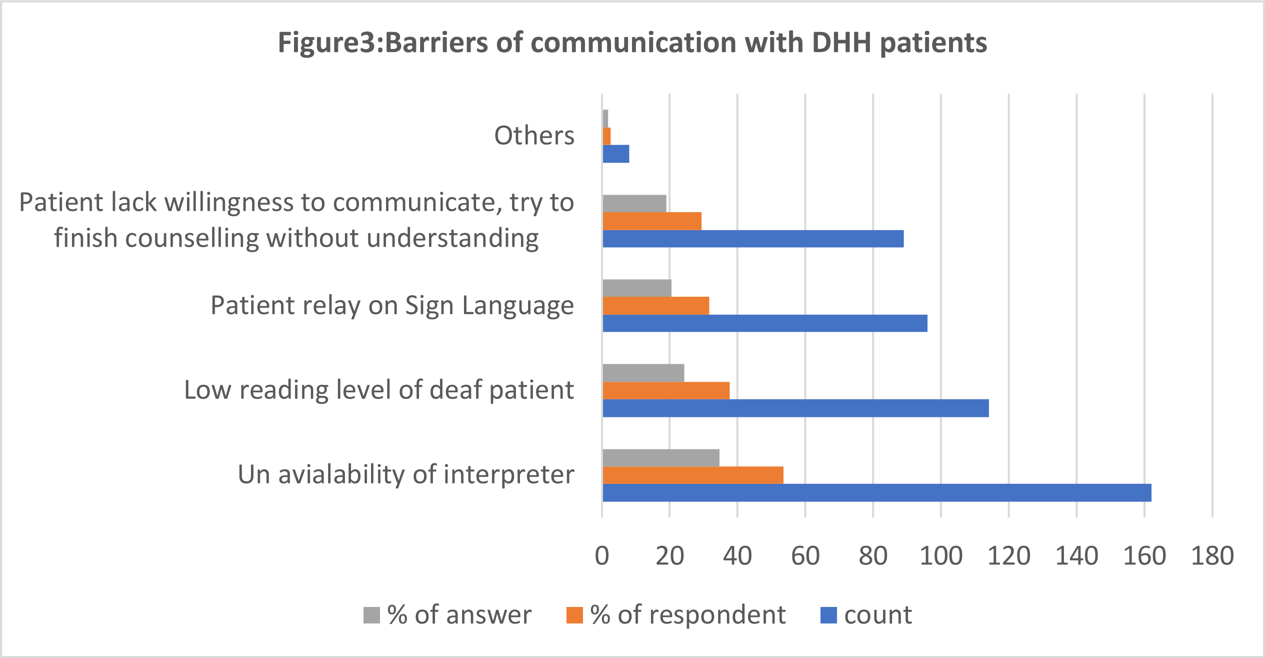

* lack of pharmacists' knowledge about sign language, patients having multiple difficulties and did not have a caregiver , difficulty in reading lips of female pharmacists because of their face cover.
